# Multi-Level Clustering of Dynamic Directional Brain Network Patterns and Their Behavioral Relevance

**DOI:** 10.3389/fnins.2019.01448

**Published:** 2020-02-06

**Authors:** Gopikrishna Deshpande, Hao Jia

**Affiliations:** ^1^Department of Electrical and Computer Engineering, AU MRI Research Center, Auburn University, Auburn, AL, United States; ^2^Department of Psychology, Auburn University, Auburn, AL, United States; ^3^Center for Neuroscience, Auburn University, Auburn, AL, United States; ^4^Center for Health Ecology and Equity Research, Auburn, AL, United States; ^5^Alabama Advanced Imaging Consortium, Birmingham, AL, United States; ^6^Department of Psychiatry, National Institute of Mental Health and Neurosciences, Bengaluru, India; ^7^School of Psychology, Capital Normal University, Beijing, China; ^8^Key Laboratory for Learning and Cognition, Capital Normal University, Beijing, China; ^9^Department of Automation, College of Information Engineering, Taiyuan University of Technology, Taiyuan, China

**Keywords:** dynamic brain connectivity, resting state fMRI, effective connectivity, clustering, behavioral relevance, human connectome

## Abstract

Dynamic functional connectivity (DFC) obtained from resting state functional magnetic resonance imaging (fMRI) data has been shown to provide novel insights into brain function which may be obscured by static functional connectivity (SFC). Further, DFC, and by implication how different brain regions may engage or disengage with each other over time, has been shown to be behaviorally relevant and more predictive than SFC of behavioral performance and/or diagnostic status. DFC is not a directional entity and may capture neural synchronization. However, directional interactions between different brain regions is another putative mechanism by which neural populations communicate. Accordingly, static effective connectivity (SEC) has been explored as a means of characterizing such directional interactions. But investigation of its dynamic counterpart, i.e., dynamic effective connectivity (DEC), is still in its infancy. Of particular note are methodological insufficiencies in identifying DEC configurations that are reproducible across time and subjects as well as a lack of understanding of the behavioral relevance of DEC obtained from resting state fMRI. In order to address these issues, we employed a dynamic multivariate autoregressive (MVAR) model to estimate DEC. The method was first validated using simulations and then applied to resting state fMRI data obtained in-house (*N* = 21), wherein we performed dynamic clustering of DEC matrices across multiple levels [using adaptive evolutionary clustering (AEC)] – spatial location, time, and subjects. We observed a small number of directional brain network configurations alternating between each other over time in a quasi-stable manner akin to brain microstates. The dominant and consistent DEC network patterns involved several regions including inferior and mid temporal cortex, motor and parietal cortex, occipital cortex, as well as part of frontal cortex. The functional relevance of these DEC states were determined using meta-analyses and pertained mainly to memory and emotion, but also involved execution and language. Finally, a larger cohort of resting-state fMRI and behavioral data from the Human Connectome Project (HCP) (*N* = 232, Q1–Q3 release) was used to demonstrate that metrics derived from DEC can explain larger variance in 70 behaviors across different domains (alertness, cognition, emotion, and personality traits) compared to SEC in healthy individuals.

## Introduction

The view that human brain functions as a coordinated system with functional segregation and integration between different regions has been corroborated and widely accepted (Friston et al., 1993; Greicius et al., 2009; Guye et al., 2010; Rogers et al., 2010). A bulk of this evidence at the macro-level comes from connectivity studies based on non-invasive resting state functional magnetic resonance imaging (fMRI). Functional connectivity (FC) is a term used to describe measures of synchronous, non-directional, correlation of inter-regional brain activity in time. Effective connectivity (EC), on the other hand, is a term used to describe measures of directional relationships between brain activity in different brain regions (Friston, 1994; Deshpande et al., 2011b; Valdes-Sosa et al., 2011; Deshpande and Hu, 2012). Previous studies mainly investigated static FC (van de Ven et al., 2004) and EC (Roebroeck et al., 2005; Stilla et al., 2007, 2008; Deshpande et al., 2009b, 2013; Hampstead et al., 2011; Sathian et al., 2011, 2013; Liang et al., 2014) characteristics, assuming that connectivity is stationary in time, and the relevance of FC (Greicius et al., 2007; Kelly et al., 2008; Han et al., 2013) and EC (Liao et al., 2010; Inman et al., 2012) to behavior and brain disorders. Further, some studies have reported that static EC relationships at rest represent a mode of communication between brain regions whose activities are not synchronized (Deshpande et al., 2011b), and hence, both FC and EC taken together, provide complementary characterizations of brain connectivity at rest. However, recent evidence points to the fact that resting state FC is not stationary in time and consequently an array of methods have been proposed to capture dynamic variations in FC (Deshpande et al., 2006; Sato et al., 2006; Britz et al., 2010; Chang and Glover, 2010; Sakoðlu et al., 2010; Chang et al., 2013a, b; Majeed et al., 2011; Cribben et al., 2012; Dimitriadis et al., 2012; Fornito et al., 2012; Handwerker et al., 2012; Hutchison et al., 2012, 2013; Rack-Gomer and Liu, 2012; Tagliazucchi et al., 2012; Keilholz et al., 2013; Lee et al., 2013; Leonardi et al., 2013). This raises the possibility that dynamic alterations in resting state EC cannot be ignored and needs to be investigated. However, to the best of our knowledge, there has been scant literature on dynamic EC of resting state fMRI (but see Jin et al., 2017; Zhao et al., 2017; Rangaprakash et al., 2018), and most investigations of dynamic EC have focused on task-based fMRI (Sato et al., 2006; Havlicek et al., 2010; Grant et al., 2014, 2015; Lacey et al., 2014; Wheelock et al., 2014; Hutcheson et al., 2015; Feng et al., 2016, 2018; Hampstead et al., 2016; Wang et al., 2017; Ramaihgari et al., 2018; Rao et al., 2018).

In order to holistically characterize connectivity in resting state brain networks, it is necessary to employ regions across the whole brain to conduct connectivity analysis. In this regard, there exists many studies exploring whole-brain static FC (Shirer et al., 2012; Zeng et al., 2012), and several others exploring whole-brain dynamic FC (Allen et al., 2013; Leonardi et al., 2013; Syed et al., 2017, 2019; Zhao et al., 2018) using principle component analysis (PCA) or independent component analysis (ICA)-based methods as well as using full pairwise connectomes instead of seed-based analysis. However, whole-brain EC analyses are less numerous due to associated challenges such as computational complexity and model discovery (Stephan and Roebroeck, 2012). For example, methods such as dynamic causal modeling (Friston et al., 2003) and structural equation modeling (McIntosh and Gozales-Lima, 1994; Zhuang et al., 2005) impose restrictions on the number of regions (but see whole brain DCM based on sparsity constraints: Frassle et al., 2018) that can be included in the model. In addition, it becomes difficult to formulate *a priori* hypotheses regarding connections between all brain regions (Lohmann et al., 2012), which are required by these methods. Therefore, data-driven approaches have become popular while investigating EC between large numbers of brain regions. One such model is the multivariate autoregressive (MVAR) model, which is used to capture time-lagged Granger causal influences between brain regions (Granger, 1969; Geweke, 1982; Deshpande et al., 2008, 2009a,b, 2010a,b, 2011a,b; Krueger et al., 2011; Wen et al., 2013). It has been previously demonstrated that the precision of the MVAR model increases when more variables containing information regarding the underlying system are included in the model (Kus et al., 2004). Yet, to estimate the parameters of an MVAR model fit using all voxel time series in the brain would require more data (in terms of the length of the time series and number of subjects) as well as require very large computational power (in terms of time and memory requirements) that may make it practically impossible. Besides, an MVAR model with too many voxel time series as regressors is ill-conditioned and highly sensitive to noise. To address this issue, dimensionality reduction is often employed and an exemplary work employing whole-brain regions/voxels for static EC can be found in Wu et al. (2013). These challenges become even more acute while computing whole-brain dynamic EC. In this present paper, we address these challenges by adopting a dynamic MVAR for characterizing dynamic EC in combination with a dimensionality reduction strategy based on multi-level clustering of dynamic EC patterns across spatial location, time, and subjects.

Clustering of dynamic EC patterns over time is motivated by evidence from dynamic FC analysis with fMRI and EEG data which show that the synchronized blood oxygenation level dependent (BOLD) signal fluctuations over the brain organize into a finite number of configurations alternating with each other in time (Britz et al., 2010; Chang and Glover, 2010; Musso et al., 2010; Li et al., 2014). One principled approach to find dynamic FC configurations which are quasi-stable for a certain period of time can be found in Li et al. (2014). This follows from similar quasi-stable scalp voltage configurations, called microstates, obtained from agglomerative clustering of EEG data (Britz et al., 2010; Musso et al., 2010). Such approaches assume that a single FC configuration exists across the entire brain at any given time instant. Additionally, they also assume that the dynamics of connectivity is essentially due to the brain changing from one across-the-brain connectivity configuration to another. In this work, we investigated this issue with regard to dynamic EC. To find the dynamic EC configurations over time, we employed clustering of EC patterns over time using the adaptive evolutionary clustering (AEC) algorithm (Jia et al., 2014). Specifically, we performed simulations in order to demonstrate the efficacy of the temporal clustering for capturing dynamic EC regimes and subsequently, we applied it to resting state fMRI data.

**FIGURE 1:**
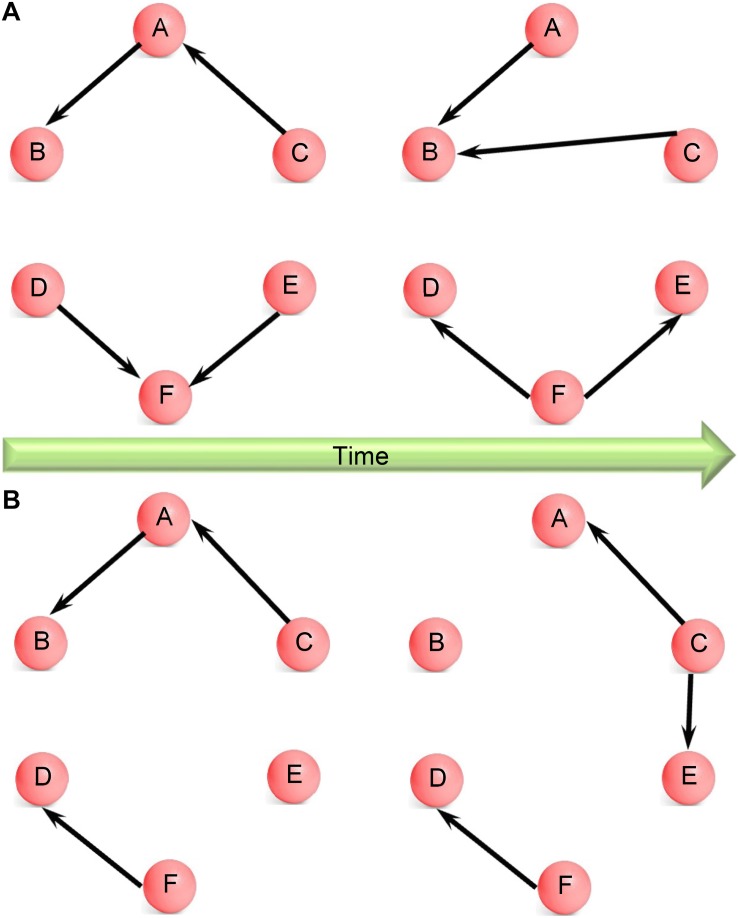
**(A)** Schematic illustrating changing network configuration over time wherein all nodes are part of the same network, only the directional connections between them change with time. For example, nodes A, B, and C are part of the same network at both time instants, but the connections between them change from the first to the second time instant. Here, C influences A at the first time instant whereas this is absent at the second time instant, when C influences B. Yet, at both time instants, nodes A, B, and C are a single connected component. **(B)** Schematic illustrating changing network configuration over time wherein both directional connections between nodes and the networks themselves are changing with time. For example, nodes A, B, and C are part of the same network at the first time instant; however, at the second time instant, nodes A, C, and E are part of the same network and node B is left out. Here, no connections between C and E changes to a directional connection from C to E. Therefore, both connections and network configurations change with time.

The amount of information obtained from the assessment of dynamic EC over the whole brain can be quite large. Hence, it has been often difficult to interpret the underlying neuroscientific meaning (Chang and Glover, 2010). Some previous studies showed snapshots of dynamic FC at various points during the experiment obtained by using different window lengths (Handwerker et al., 2012; Lee et al., 2013) or template pattern matching (Majeed et al., 2011). Although a very good exploratory technique, the results, and interpretation from such an approach can be subjective, depending on the window length and frames chosen. Time–frequency analysis can overcome this difficulty by projecting dynamics of connectivity onto time–frequency plane (Chang and Glover, 2010) using wavelet-based methods. But the information obtained this way is difficult to interpret. Besides, the approaches based on agglomerative clustering (Britz et al., 2010; Musso et al., 2010) (used in microstate analysis) and principal component analysis (Leonardi et al., 2013) make an assumption that the connectivity networks may have different weights in spatial or temporal domains, but the spatial configuration of connectivity networks themselves do not change with time. This assumption may suffer from loss of generality. Therefore, in this paper, we propose an approach which does not make such an assumption. The AEC algorithm yields time-varying clustering configurations wherein the clusters (networks) themselves could change over time, as well as the ECs in each network. For instance, there are two networks shown in Figure 1A. Each network has the same nodes over time, so the network itself does not change, only the connections between nodes change with time. By contrast, the case shown in Figure 1B is more general wherein both connections between nodes and the networks themselves change with time, i.e., the networks have different nodes at different time instants. The clustering algorithm used in this work is capable of modeling the more general case. With this merit, the proposed method is likely to hold promise in a variety of situations.

In order to determine EC configurations between brain regions which may be reproducible across different time instants within a given run, as well as across different subjects, two additional levels of clustering were employed across all fMRI runs and subjects, one level for determining most reproducible spatial configurations across time instants and another level for determining such consistent patterns across subjects. As we have shown in the case of static connectivity (Deshpande et al., 2011b), absence of significant synchronous connectivity does not imply the absence of brain connectivity, rather, such regions could be communicating via non-synchronous relationships (such as causality) that may be captured via EC. Our work can complement existing FC studies, since both synchronization and causality are established mechanisms of brain connectivity and one needs to assess both measures in order to gain a complete understanding of brain connectivity (Deshpande and Hu, 2012).

Once group-level dynamic EC patterns have been found using the proposed multilevel clustering approach, we tested the hypothesis that such dynamic EC patterns may be behaviorally salient. Specifically, we tested the hypotheses that (i) greater temporal variability of EC increases the adaptability and efficiency of brain networks, leading to better behavioral performance, and (ii) dynamic EC may better predict behavior than their static counterparts. These hypotheses were motivated by evidence in their favor in the context of dynamic FC as in our previous study (Jia et al., 2014). In order to test these hypotheses, we used resting-state fMRI and behavioral data from the Human Connectome Project (HCP) to correlate dynamic and static EC-based metrics with behavioral data in various domains, such as alertness, emotion, cognition, and personality traits.

## Materials and Methods

### Data

#### Data Acquisition and Pre-processing (Cohort-1)

Resting-state fMRI data were acquired from a 3T Siemens Verio scanner at the Auburn University MRI Research Center from 21 healthy adults (aged 29.68 ± 11.06 years, nine females). Informed consent was obtained from all subjects after explaining and reviewing detailed written information about the study protocol, which was approved by the IRB of Auburn University. In all experiments, subjects were lying at rest during the scan with eyes open, and they were instructed to be awake and let their mind wander and not think about anything in particular. After the scan, all subjects confirmed adherence to these guidelines. T2^∗^-weighted echo planar imaging with the following parameters were used for fMRI data acquisition: 1000 volumes (time points) per run, in-plane matrix of 64 × 64, 16 axial slices covering the entire cerebral cortex, field of view (FOV) = 225 mm × 225 mm, flip angle (FA) = 90°, TR (repetition time)/TE (echo time) = 1000 ms/29 ms, in-plane voxel size = 3.5 mm × 3.5 mm, slice thickness of 5 mm with 1.25 mm gap. Standard anatomical MPRAGE data were also acquired from each subject for spatial normalization. Functional MRI preprocessing was performed using Data Processing Assistant for Resting-State fMRI software (DPARSF) (Yan and Zang, 2010) and included slice timing correction, rigid body registration, normalization to MNI template with resampling to 2 mm × 2 mm × 2 mm resolution, spatial smoothing with 4 mm × 4 mm × 4 mm full width at half magnitude (FWHM) Gaussian kernel, 0.01–0.1 Hz band pass filtering, detrending of mean and linear trend, and regressing out of nuisance covariates such as physiological artifacts and residual motion using aCompCor (Muschelli et al., 2014). Then, the 190-region version of the CC200 brain atlas (Craddock et al., 2012) was used as the reference brain parcellation template. We extracted the mean time series from 164 cerebral regions for subsequent use (26 regions of the CC200 template belonging to the cerebellum were not considered because our field of view covered only the cerebrum; this was done in order to reduce the TR which would be beneficial for EC analysis).

#### Behavioral and Individual Difference Measures (Cohort-2)

The subjects from the first cohort described above were used for demonstration of the proposed method in experimental data. However, we did not have detailed behavioral phenotyping of these subjects in order to demonstrate the behavioral relevance of the dynamic EC patterns. Therefore, we also used a second cohort consisting of resting-state fMRI data obtained from the HCP (*N* = 232, Q1–Q3^1^). These data underwent the same pre-processing pipeline as the first cohort. Behavioral measurements from same subjects have also been used in order to test the relative ability of static and dynamic EC for explaining behavior. HCP mainly measures behavioral data developed for NIH Toolbox Assessment of Neurological and Behavioral function^2^ and some other measurements that are not covered by the NIH toolbox. The behavioral measures employed in this work belonged to the following domains: alertness, cognition, personality, and emotion. Notably, motor and sensory functions were not included in our analysis since they may be more relevant to while examining task data.

### Analysis Methodology

#### Dynamic Effective Connectivity Model

The traditional static formulation of the multivariate vector autoregressive (MVAR) model is shown below

(1) $$Y\,\left(t\right)=B+\sum_{m=1}^{p}K\,\left(m\right)\cdot Y\,\left(t-m\right)+\text{N}\,\left(t\right)$$

where ***Y***(*t*) = [*y*_1_(*t*) *y*_2_(*t*) ⋯ *y*_*l*_(*t*)] is a vector autoregressive process including *l* individual univariate processes [in our scenario, *l* is 164, the number of regions spanning the cerebrum in the CC200 brain parcellation atlas (Craddock et al., 2012)], ***B*** is the intercept vector representing the non-zero mean component, *m* denotes the time lag (in terms of TRs), ***K***(*m*) corresponds to the model coefficient matrix, *p* is the model order, and ***N***(*t*) is the vector noise process. Since during preprocessing, the data were detrended and mean centered, ***B*** vanished. Then, the Granger causality (static version) representing direct causal influences from region *i* to region *j* is formulated as below.

(2) $$G_{i\,j}=\sum_{m=1}^{p}{\left[k_{i\,j}\,\left(m\right)\right]}^{2}$$

where each *k*_*ij*_, *i*, *j* = 1:*l*, is one entry of matrix ***K*** with row number being *i* and column number being *j*. ***K*** is determined in the least square sense. The order *p* of this MVAR model can be determined according to Bayesian Information Criterion (BIC) (Schwartz, 1978; Roebroeck et al., 2005) (in our data, *p* = 1 since we are interested in relationships with lags equal to or less than a TR). Then, estimation of EC dynamics was obtained through dynamic Granger causality (DGC) which differs from static Granger causality in terms of coefficient matrix ***K***, which is allowed to vary over time. Then the MVAR model in Eq. 1 changes to Eq. 3 accordingly.

(3) $$Y\,\left(t\right)=B\,\left(t\right)+\sum_{m=1}^{p}K\,\left(m,t\right)\cdot Y\,\left(t-m\right)+\text{N}\,\left(t\right).$$

Note here that both coefficient matrix ***K***(*m*, *t*) and ***B***(*t*) are a function of both lag *m* and time *t*. The DGC metric is then formulated as below.

(4) $${\text{DGC}}_{i\,j}\,\left(t\right)=\sum_{m=1}^{p}{\left[k_{i\,j}\,\left(m,t\right)\right]}^{2}.$$

Model coefficients ***K***(*m*, *t*) were estimated based on a previously used procedure which utilizes Kalman filtering (Arnold et al., 1998). For Kalman updating of coefficient matrix ***K***(*m*, *t*), we imported a parameter called forgetting factor, *F*, to control the way of updating. 1−*F* is actually the weighting of recent past Kalman estimate of ***K*** in the current estimate of ***K.*** The weighting for most recent past ***K*** is 1−*F*, and exponentially decreases when moving backward. This is due to the consideration of boosting estimation stability and *F* was optimized by minimizing the variance of estimated error energy as follows (Schlogl et al., 2000; Havlicek et al., 2010).

(5) $$F=\arg\{\min\left(var\left(\tilde{N}{\left(t\right)}^{2}\right)\right\}$$

where $\tilde{N}\,\left(t\right)$ is the estimate of ***N***(*t*) and “var” is the variance operation over time.

In order to estimate a reasonable initial condition for the Kalman filter, we used the following procedure. The Kalman filter coefficients were randomly initialized and updated coefficients were obtained from the first run of the first subject which were in turn used as initial conditions for the following run/subject. Using this procedure iteratively, Kalman coefficients which were updated using the entire subject sample were obtained. This represented Kalman coefficients of the entire group as a whole. This group value was used as the initial condition for all runs/subjects and DGC values were re-estimated at the individual subject level. This procedure ensured that for each subject, the Kalman filter coefficients were initialized to the same value which was representative of the group average, which helped in relatively quick convergence. However, the DGCs obtained from the first 50 time points were discarded before being input into the clustering algorithm for the following reasons. First, initial time points in fMRI time series are routinely discarded to allow the MR signal to achieve T1 equilibration. Second, even with a group-averaged Kalman coefficients as the starting condition, the Kalman filter needed time to converge to ground truth connectivity as shown by our simulations (see simulation results for illustration).

#### Clustering

**FIGURE 2:**
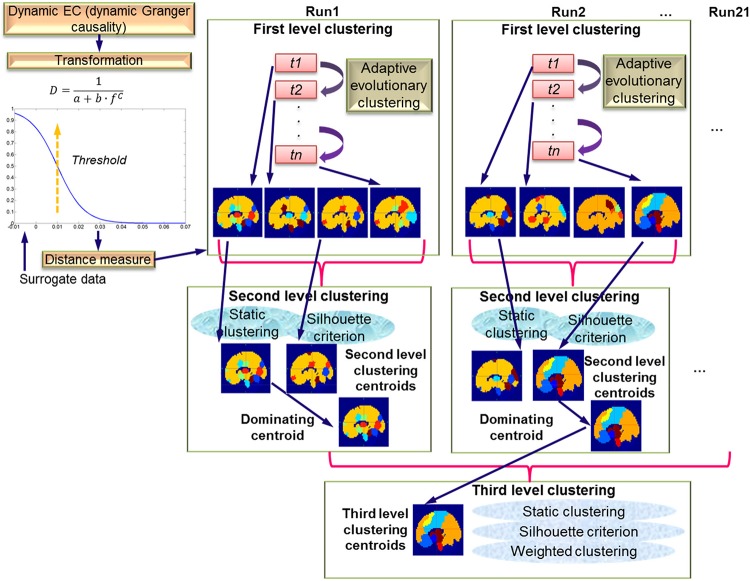
Schematic of the three-level clustering procedure. The top left part shows the transformation of dynamic EC measure (DGC) to a distance measure. Surrogate data were used to determine significant connections. The top right part shows first level clustering using AEC across time instants t1 to tn. The results of the first level clustering are fed into second level clustering, which is static. Here, silhouette criterion is used to determine number of clusters. The dominating centroids from the second level are fed to the third level clustering which is also static and uses the silhouette criterion along with weighted clustering.

The DGC matrix calculated via the above procedures was of size *X* × *X* × *Y* × *Z* where *X* = 164, the number of cerebral regions, *Y* = 950, the number of TRs in DGC calculation (each run had a total of 1000 TRs, first 50 TRs discarded), and *Z* = 21, the number of runs/subjects. So, for each run, at each time instant, the EC between all pairs of regions had a dimension of *X* × *X*. This DGC matrix was then fed into the AEC algorithm (Xu et al., 2013). This algorithm dynamically clustered all 164 regions according to their distances (the distances were transformed from the EC metric, for details see the section “First Level Clustering”) at every time instant. Likewise, a forgetting factor was introduced to control the impact of the recent past of clustering results on the current calculation, with the purpose of enhancing stability of the clustering operation. This clustering is termed first level clustering and is described in detail in the following section. As mentioned in the section “Introduction,” this clustering strategy can accommodate not only the changes of EC between nodes in a given network, but also the network configuration itself in terms of the nodes that may make up the network (Figure 1B). The first level clustering result revealed time-varying brain network configurations which were then fed to second level clustering as members in order to determine the distribution and consistency of the first level configurations across different time instants across a given run. Last but not the least, the dominating second level patterns from all runs were identified and used as members for the third level clustering. Resultantly, by the third level clustering, the dominant and consistent brain EC network patterns across all runs and subjects were identified. A graphical presentation of the hierarchy of three levels of clustering is given in Figure 2.

#### First Level Clustering

The first level clustering was implemented using the AEC algorithm employing a distance measure computed from the DGC matrix. A reasonable assumption is that the higher the absolute value of DGC, the closer the two regions are in feature space. Also noteworthy is that the DGC value between regions cannot be utilized directly as distances between regions for clustering. We know that the distance is inversely proportional to the closeness between regions, but the DGC metric is proportional to the closeness. Next, distance measure is greater or equal to zero, being zero only when it is measured from one region to itself. However, DGC metrics have both positive and negative values, and the diagonal entries of DGC matrix measuring auto-DGC are not zero. Moreover, DGC matrices are not symmetric, i.e., the distance from one region to another is not equal to the other way around, violating the condition of reciprocity required of any distance measure.

In order to convert DGC values into a distance measure, we devised a transformation algorithm as described below. We represent the DGC from region *i* to region *j* by DGC*_*ij*_*, and the other way around is DGC*_*ji*_*. DGC was transformed as shown below to meet the non-negative and reciprocity requirements.

(6) $$C={\left({\left|D\,G\,C_{i\,j}\right|}^{m}+{\left|D\,G\,C_{j\,i}\right|}^{m}\right)}^{1/n}$$

where *m* and *n* determine the characteristics of this transformation. According to Eq. 6, *C* will increase along with the increase of either DGC*_*ij*_* or DGC*_*ji*_*. We chose *m* = 2 and *n* = 1 in this work, as such is usually employed for a second order matrix norm. If *n* is relatively large compared to *m*, then *C* would not be sensitive to the change of DGC, thus cannot distinguish significant connectivity from trivial ones. If *m* is relatively large compared to *n*, the result will be sensitive to noise. Therefore, *m* = 2 and *n* = 1 seemed to be an optimal choice. Next, we used a reversed “S” shaped function applied to *C* to meet the requirement of a monotonically decreasing transformation:

(7) $$D=\frac{1}{a+b\cdot f^{C}}$$

where *a*, *b*, and *f* are control parameters determining the behavior of this function. After this transformation, the significant connectivity between two regions are highlighted while non-significant ones are not. In order to find the boundary between significant and insignificant connectivities, we employed the method of surrogate data (Theiler et al., 1992; Deshpande et al., 2009b). Specifically, we transformed the time series to their frequency domain representation, randomized the phase of time series from all 164 regions in the frequency domain with magnitude unchanged, reconverted the phase-randomized data into time domain signals, and then the DGC was recalculated. Since the temporal structure of time series relative to each other was destroyed due to phase randomization, the DGCs obtained belonged to a null distribution of no influence between regions. After this procedure was repeated in a Monte Carlo manner (1000 times), a statistical null distribution of insignificant DGCs was obtained for each connection. Then we applied Eq. 6 to get the null distribution of *C* and found the threshold at 95th percentile, denoted by *th*_0_. It is obvious that parameter *a* just controls the scaling of *D*, thus is trivial, and hence we set it to 1 for normalization. In this way, *D* can attain its maximum at 1/(1 + *b*) and an asymptotic minimum at 0. Assume *b* is sufficiently small such that the maximal value that *D* can reach is asymptotically equal to 1. Then it is reasonable to let *D* be 0.5 when *C* is equal to *th*_0_, and when *C* approaches 0, *D* is asymptotically equal to 1. With surrogate data, we found *th*_0_ to be equal to 0.01. To find *b*, we followed previous work (Kent et al., 1972; McDowall and Dampney, 2006). Accordingly, we restricted *D* to be no <0.8 and steepness to be <0.02 when *C* is 0. So, when *D* = 0.8, steepness is 0.02 when *C* is 0, we calculated *b* to be 0.2, and *f* as 5^100^ which were used in the following. Above sigmoid curve design aims to balance the separation of null and significant connectivity, and sensitivity to noise. The sigmoid curve design and parameters determination has been widely applied in many fields (Kent et al., 1972; McDowall and Dampney, 2006). After the transformation of Eq. 7, we let the *D*s which were from one region to itself to be zero, resulting in the final distance measure *D*_final_.

The estimated distance matrix *D*_final_ at each time point was fed into the AEC algorithm (Xu et al., 2013). With this algorithm, we did clustering over all 164 regions at one time instant, taking the clustering result at recent previous time instants into account with adaptive weighting. The weighting was calculated through a forgetting factor, which was determined by BIC (Roebroeck et al., 2005). The clustering method was chosen to be hierarchical (Joe and Ward, 1963).

The number of clusters can be chosen either based on prior information about the neurophysiological system being investigated (which is preferable when that information is available) or based on mathematical criteria such as the silhouette index (Rousseeuw, 1987) (which is preferable when no *a priori* heuristics are available). Many previous studies have reported on the appropriate number of clusters, or in other words, the number of resting state networks (RSNs). To derive an eloquent result, various methods have been tried. The most representative one is ICA-based methods. In particular, probabilistic ICA (PICA) (De Luca et al., 2006) and tensor PICA (Damoiseaux et al., 2006) are variants of ICA which has attracted a lot of attention recently. Fully exploratory network ICA (FENICA) (Schöpf et al., 2010, 2011; Kalcher et al., 2012; Wang et al., 2012) has also been shown to find consistent networks among a group which may include thousands of subjects. Besides, fuzzy clustering (Lee et al., 2012) and graph theory (Moussa et al., 2012) are also two prevailing methods to find the number of RSNs with their own merits. Except a few of the above studies which employed task-related data (Schöpf et al., 2011), most studies focus on resting-state fMRI data. However, these findings are in terms of FC, and corresponding EC results are very sparse. A data-driven pilot study conducted by Wu et al. (2013), reported six communities from resting-state EC networks. Therefore, we used six as the number of clusters in first level clustering. Also, we assume that EC networks are hierarchically organized, similar to FC networks (Kalcher et al., 2012; Lee et al., 2012). Accordingly, if the specified number of networks or clusters increases, some networks will split into sub-networks, such as into left and right lateral parts or peripheral and foveal parts, rather than reshape into a new set of networks which has no relation with the previous one. Based on this assumption, if the number of networks/clusters is heuristically specified to be 6, it may not lead to loss of generality.

#### Second Level Clustering

We performed second level clustering for characterizing consistently recurring first-level patterns over time. This aided us to answer the question about whether there exists finite number of directional brain network patterns which consistently recur in time at an individual subject level. The difference from first level clustering is that this is a static clustering along the time axis. Hierarchical method was employed as the clustering method, and the number of clusters was determined by silhouette criterion in the absence of prior heuristics.

At this stage, we devised the distance measures between first level clustering configurations at different time instants using the following strategy. For any given two first level clustering configurations, we assume the first one has *M* clusters denoted by *a*_*m*_, *m* = 1, 2, …, *M*, and the second one has *N* clusters denoted by *b*_*n*_, *n* = 1, 2, …, *N*. For each pair of clusters with one picked out from the first configuration and the other picked out from the second configuration, the number of common regions is computed and among them, the maximal one is found. For the very pair having the maximal number of common regions, all regions in them are given a uniform label. For example, suppose the 5th cluster comprised of region #1, region #2, region #3 in the first configuration and 4th cluster comprised of region #2, region #3, region #5 in the second configuration have the most common regions (regions #2 and #3), then regions #1, #2, and #3 in the first configuration and regions #2, #3, and #5 in the second configurations are given the same label ①. Next, we delete this pair and for all remaining clusters, redo the above operation, i.e., find the pair of clusters which have most common regions and give all clusters inside them the same label ②. Then we delete the second pair and redo the above operation. When the maximal number of common regions becomes zero over many iterations, we give the clusters in the first configuration a label, for example, ③ and those in the second configuration a different label, for example, ④. Then we delete this pair and redo the above operation. We iterate this procedure until no pair is left. After that, we set the initial distance measure to be zero, then we transverse all regions, if one region has different labels in the first and second configurations, the distance measure adds by 1, otherwise it does not change. So the final distance measure is actually the number of regions having different labels in the two configurations. It is self-evident that this distance measure meets the requirement of reciprocity, non-negativeness, triangular inequality, and the distance from one region to itself is zero.

After second level clustering, similar first level clustering configurations are grouped together, forming one pattern. Since a cluster can be represented by its centroid, we discuss how to represent each second level cluster centroid. The theoretical centroid is the weighted sum of first level configurations indicating that each region in the theoretical centroid owns fuzzy memberships to all first level clusters inside a given second level cluster. This centroid is very awkward to use, especially for third level clustering. Therefore, we represent the theoretical centroid by an agent which is described below. For every second level cluster, we average out all distance matrices ***D*_*i*_**s of all its members, resulting in the mean matrix ***D***_mean_. Then the first level configuration inside this second level cluster with smallest Euclidean distance to ***D***_mean_ is set as the agent (denoted by *i*_agent_) for theoretical centroid, i.e.,

(8) $$i_{\text{agent}}=\arg\left(\min\left(\sum_{r=1}^{R}\sum_{c=1}^{C}{\left(D_{i}\,\left(r,c\right)-D_{\text{mean}}\,\left(r,c\right)\right)}^{2}\right),i\right)$$

where *C* and *R* represent the number of columns and rows in the distance matrices.

#### Third Level Clustering

The second level clustering gives clustered patterns over time for each run and for each subject. The dominating second level patterns/clusters were predicated based on the histogram of second level clusters’ occurrence times. Here the occurrence times is formulated as follows. For each run, each second level cluster covered a number of first level clustering configurations, the number of which was the times this second level cluster “occurred,” so was defined as occurrence times for the given second level cluster. The histogram mentioned above was calculated over second level clusters from all runs. The threshold separating dominating, and non-dominating clusters was determined as follows. The point where the histogram value first hits zero, and the corresponding first-order derivative is also zero, is set as the threshold. In our data, this threshold was found to be at 95 (occurrence times). There was clearly a gap encompassing 95, and the majority of second level clusters had occurrence times <95, while beyond 95, second level clusters were relatively scarce and mainly distributed over the range: 100–200, 270–360, and 450–850, clearly indicating that they are dominant clusters. In order to assess the consistency of these dominating patterns across subjects, we performed third level clustering.

To calculate the distance measure between dominating second level centroid agents, it is inappropriate to adopt the strategy adopted in second level clustering since each centroid has a weight that we need to account for, i.e., the occurrence times of the pattern it represents. Therefore, a weighted clustering was adopted and is described as follows. To represent each dominating second level centroid agent in feature space, we vectorized its distance matrix (as illustrated in Eqs. 7 and 8) without recruiting diagonal entries (diagonal entries are all zeros and hence useless), such that the resulting vector is the representative in feature space. Next, the weighted *K*-means clustering was performed over these vector representatives, resulting in several patterns at the third level. As before, the number of clusters were optimized using the silhouette criterion. Each third level cluster’s theoretical centroid is represented by its agent since the regions in the theoretical centroid have fuzzy memberships to dominating second level clusters. The agent has smallest Euclidean distance to the theoretical centroid in terms of distance. The three-level clustering procedure for dynamic EC described above is illustrated in Figure 2.

#### Potential Correlates With Real World Functionalities

Through the third level clustering, the brain’s EC network patterns which were dominant and consistent across all subjects were obtained. To interpret the neural connotations of these patterns, we related them to real world cognitive functionalities using the Brainmap Sleuth search engine (Brainmap.Org, 2019). Specifically, for a given third level centroid agent, the 164 regions inside the cerebrum were divided into six clusters. It should be noted that one of the six clusters (networks) was trivial because it included all other brain regions which were not present in the five other clusters. This is because the clustering algorithm partitions all members, and if the five networks are definitive networks, the 6th one will include every other member not inside the five networks. The trivial cluster can be separated from five other networks by visual inspection since it embodies the most regions. As such, for each of the five definitive networks, we used the list of regions it included as input to Brainmap Sleuth search engine to find functional that those regions/networks may be engaged in based on previous literature. The Brainmap Sleuth search engine allows this kind of reverse inference to me made in a mathematically principled way.

#### Simulations

Simulations were performed in order to validate the proposed method for calculating DGC and the efficacy of subsequent clustering using the AEC algorithm. We simulated time series with a total length of 1000 time points from 12 regions using an autoregressive model as given below:

(9) $$Z\,\left(t\right)=\sum_{m=1}^{p}K_{m}\cdot Z\,\left(t-m\right)+N\,\left(t\right)$$

where ***Z***(*t*) denotes the vector of signals from multiple regions, ***K****_*m*_* is the regression coefficient matrix, and ***N***(*t*) represents noise term with covariance matrix **Cov**, which has autocorrelation coefficients normalized to 1. The order *p* is chosen to be 1. Then, three scenarios are used:

1. **Cov** was identity matrix so as to remove the effect of instantaneous correlation. The 12 time series were divided into four clusters each having three members (regions 1, 2, and 3 were in one cluster, regions 4, 5, and 6 were in one cluster, regions 7, 8, and 9 were in one cluster, and regions 10, 11, and 12 were in one cluster). Thus, ***K***_1_ had block structure with 3 × 3 blocks on the diagonal, but the causality coefficients were constant over time. Each non-zero element in ***K***_1_ was selected such that ***K***_1_ had all eigenvalues within the unit circle with all diagonal terms being negative. This ensured that the simulated time series were stable and its power spectral energy was concentrated in the low frequency band, in accordance with the fact that the signal of interest in experimental fMRI data lies in the low frequency band.
2. **Cov** was still an identity matrix, and 12 time series were divided into the same four clusters as in (i). As before, ***K***_1_ had block structure with 3 × 3 blocks on the diagonal, all eigenvalues within the unit circle, and all diagonal terms being negative. But each non-zero entry in ***K***_1_ was oscillating over time sinusoidally with period equal to 200π and randomized phases. The extent of this sinusoidal oscillation was bounded under the consideration of maintaining the stability of time series.
3. **Cov** was still an identity matrix, and ***K***_1_ had all eigenvalues within the unit circle and all diagonal terms being negative. Initially, we set ***K***_1_ to have a block structure with 3 × 3 blocks on the diagonal and the same four clusters as in (i) and (ii). But after every 200 time points, ***K***_1_ was circularly shifted by one column and one row aiming to change the cluster belongingness of each region. For example, from time point 1 to 200, regions 1, 2, and 3 belong to the same cluster (indicating they are inter-connected), regions 4, 5, and 6 belong to the same cluster, regions 7, 8, and 9 belong to the same cluster, and regions 10, 11, and 12 belong to the same cluster. But from time point 201 to 400, regions 2, 3, and 4 belong to the same cluster, regions 5, 6, and 7 belong to the same cluster, …, and regions 11, 12, and 1 belong to the same cluster. Then from time point 401 to 600, region 3, 4, and 5 belong to the same cluster, and so on.

For each scenario listed above, the simulation was conducted 1000 times to get a group of simulated MVAR processes, and then the statistics of the DGCs were obtained.

#### Behavioral Relevance of Dynamic EC and Static EC

While the previous section described analyses of Cohort-1, we now describe the analysis procedure employed for Cohort-2. We used variance of dynamic EC as the metric of EC dynamics, and the absolute value of static EC as the metric of EC strength across the run. We input these two metrics into a GLM as explanatory variables and behavioral scores as dependent variables, as is given below:

(10) $$B_{i,j}=\alpha_{i,j}\cdot {\text{DEC}}_{i,j}+\beta_{i,j}\cdot {\text{SEC}}_{i,j}+\varepsilon_{i,j}$$

where *i* indexes different behavioral tests, *j* indexes the ECs between different pairs of regions, *B*_*i,j*_ is a vector of behavioral scores for all subjects, DEC*_*i,j*_*, and SEC*_*i,j*_* are vectors of corresponding dynamic/static EC metrics for all subjects. α*_*i,j*_*, β*_*i,j*_* are their coefficients, respectively, and ε*_*i,j*_* are residuals. It should be noted that we had two runs and one behavior score for each of the 44 subjects, so each subject’s behavioral score was used twice in this GLM. The coefficients obtained from this GLM were tested for statistical significance using a *z*-test. A Bonferroni-corrected *p*-value threshold of *p* = 0.05/70 = 0.00071 (70 is the number of behavioral tests) was used in this test. Then, the variance explained in this GLM by each metric was calculated. For example$\,\bar{{\left(\alpha_{i,j}\cdot {\text{DEC}}_{i,j}\right)}^{2}}$ is the variance explained by dynamic effective connectivity (DEC). The overline denotes mean operation over all *i*s and *j*s. And $\,\bar{{\left(\alpha_{i,j}\cdot {\text{DEC}}_{i,j}\right)}^{2}}/\left(\bar{{\left(\alpha_{i,j}\cdot {\text{DEC}}_{i,j}\right)}^{2}}+\bar{{\left(\beta_{i,j}\cdot {\text{SEC}}_{i,j}\right)}^{2}}\right)$ represents the relative percentage of variance explained by DEC.

## Results

### Simulations

**FIGURE 3:**
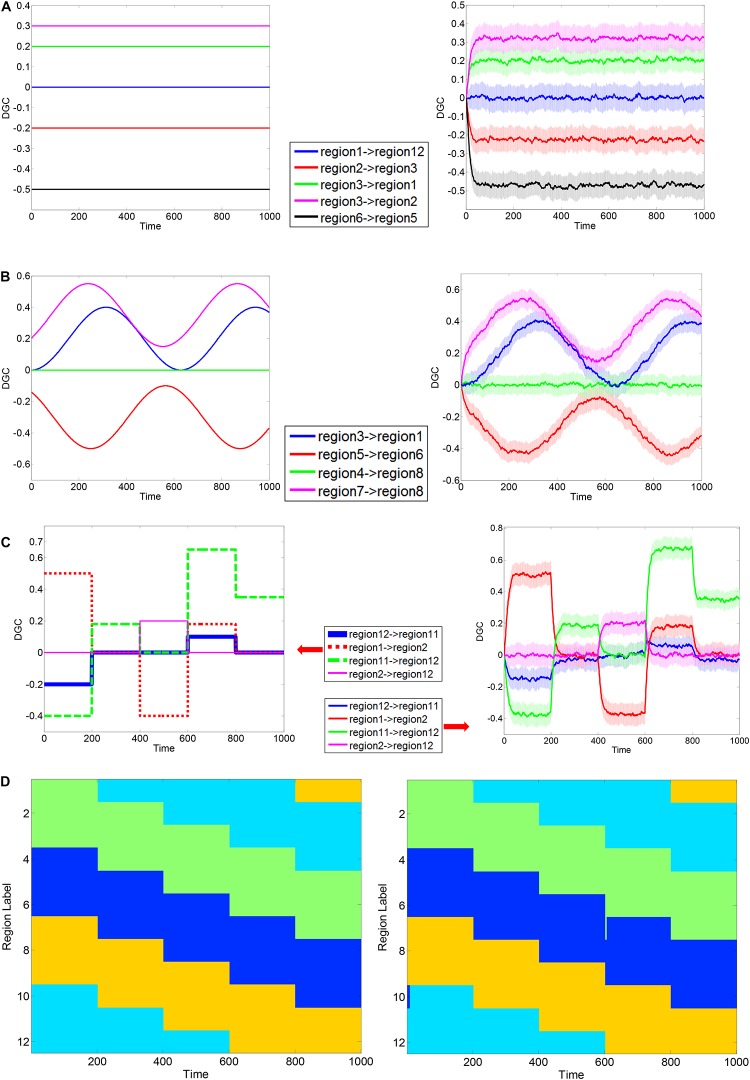
Exemplary simulation result for dynamic Granger causality and first level AEC clustering. MVAR processes of 12 regions were simulated, with a length of 1000 time points. Three scenarios were used to corroborate the validity of formulated DGC. Exemplary ground truth causality of scenario (i) is shown in panel **(A)** **Left**, and corresponding mean ± standard deviation (std) of calculated DGCs is shown at **Right**. Color bands extend from mean–std to mean + std with mean values at the center. Below is same. Exemplary ground truth causality of scenario (ii) is shown in panel **(B)** **Left**, and corresponding mean ± standard deviation (std) of calculated DGCs is shown at **Right**. Exemplary ground truth causality of scenario (iii) is shown in panel **(C)** **Left**, and corresponding mean ± standard deviation (std) of calculated DGCs is shown at **Right**. Exemplary ground truth clustering pattern corresponding to scenario (iii) is shown in panel **(D)** **Left** and corresponding clustering result estimated using AEC algorithm is shown at **Right**. Regions rendered the same color belong to the same cluster.

The simulation results are shown in Figure 3. In each part-figure in Figure 3, the simulated ground truth of DGCs are shown on the left and the estimated DGCs using the dynamic MVAR model employed in this work are shown on the right. The estimated DGCs converged to the ground truth quickly, and suitably responded to dynamic variations in ground truth DGC as shown in Figures 3B,C. Also, the regions belonging to different clusters had nearly zero causality, such as region 1 → region 12 in Figure 3A and region 4 → region 8 in Figure 3B, indicating no false positives. The standard deviation of estimated DGCs over all instantiations of the AR process was modest, indicating good fidelity. Figure 3D presents a representative realization of first level clustering using AEC algorithm for simulated DGCs in scenario (iii). Along the time axis, regions rendered the same color belong to the same cluster. It can be seen that in Figure 3D, the AEC first level clustering clearly separates the 12 regions into clusters with correct memberships in a time-varying manner. In summary, the simulations demonstrate that the proposed DGC model qualifies for tracking true dynamic ECs, and the true time-varying clustering patterns can be reliably reproduced by the AEC algorithm (first level clustering).

### Experimental Data (Cohort-1)

**FIGURE 4:**
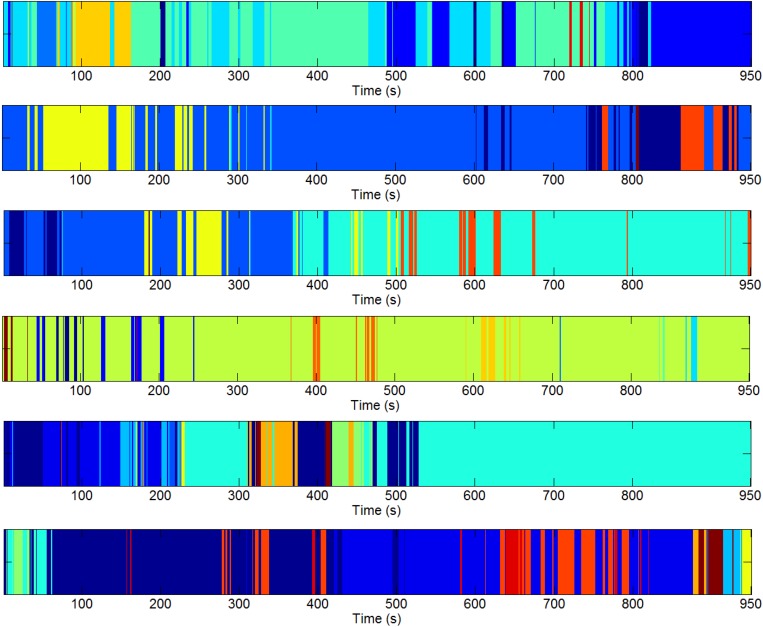
Exemplary second level clustering patterns over time axis from six runs. Along each bar, each color represents one second level cluster and the time instants it occupies indicate the first-level configurations at these time instants belong to it. Different colors represent different second-level clusters. The number of second-level clusters for each bar is 10, 6, 6, 10, 11, 11 (from top to bottom). Please note the same colors in different runs do not mean they are of the same pattern.

**FIGURE 5:**
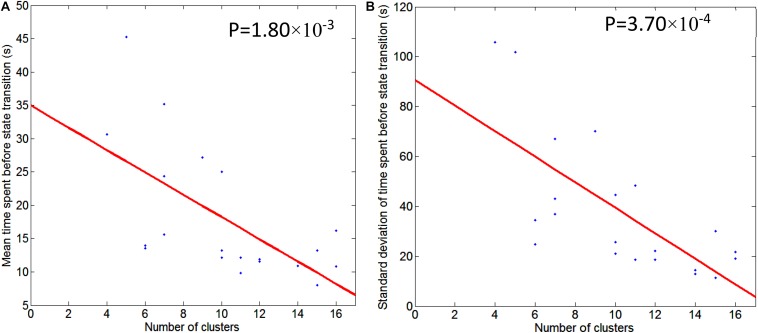
Illustration of regression of mean/std of time spent before state transition with respect to the number of second level clusters. Graph **(A)** is for mean time spent before state transition and Graph **(B)** is for standard deviation of time spent before state transition. Regression line is shown in red, and scattered dots represent data points from 21 subjects. The *p*-value for the significance of the fit using the regression line is also indicated.

**FIGURE 6:**
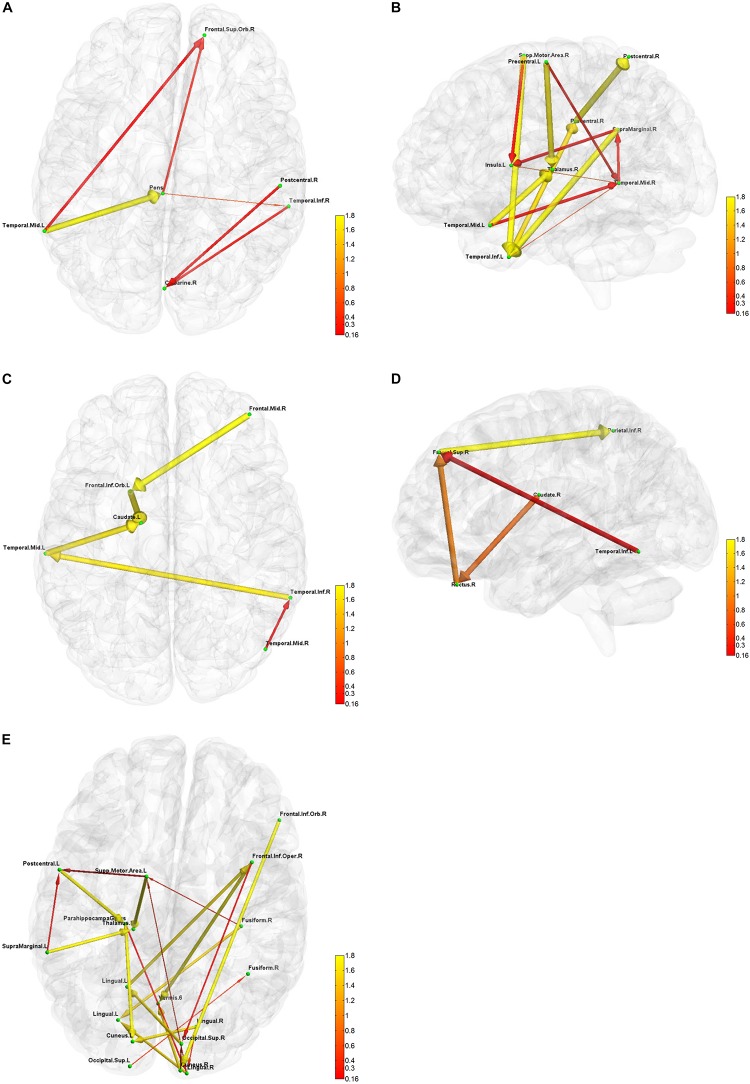
Five directional connectivity networks of the most reproducible third level clustering centroid. In each part figures **(A–E)**, green dots represent the centers of corresponding functionally homogeneous CC200 regions and arrowed paths represent directional connectivity between regions with thickness and color representing the absolute connectivity value. Autumn color map is used with red indicating small value and yellow indicating big value.

We estimated the DGC metric for each subject of pre-processed resting-state fMRI data and fed it into the three-level clustering algorithm. The results are shown in Figures 4–6 and relevant statistics are summarized in Tables 1, 2. Figure 4 presents exemplary second level clustering patterns along the time axis. Different colors represent different clusters. The number of second level clusters from the top bar to the bottom bar in Figure 4 is 10, 6, 6, 10, 11, and 11. These numbers are representative numbers of second level clusters, as reflected in Table 1. Please note that the same colors in different subjects do not mean they represent the same pattern. Table 1 presents corresponding second level clustering statistics for all subjects. By the histogram method described in the previous section, we identified one to three dominant clusters at the second level (varies from subject to subject). Critically, we can observe features similar to quasi-stability in Figure 4, i.e., each dominant pattern lasts for a period of time, during which it may swiftly switch to a few non-dominant patterns and switch back, and then switches to another dominant pattern. Certainly, dominant patterns last longer than non-dominant patterns, as expected.

**TABLE 1:** Summary of statistical characteristics of second level clusters.

**Subject**	**Number of clusters**	**Time spent (s) before any state transition (mean ± standard deviation)**	**Time spent (s) in each cluster (descending)**
#1	11	9.79 ± 18.65	350, 329, 116, 33, 31, 28, 22, 17, 13, 10, 1
#2	7	35.19 ± 67.08	777, 69, 60, 26, 13, 4, 1
#3	16	16.18 ± 21.62	456, 172, 79, 69, 62, 27, 22, 19, 11, 10, 6, 4, 4, 3, 3, 3
#4	4	30.65 ± 105.84	732, 179, 24, 15
#5	12	11.59 ± 18.61	348, 307, 112, 70, 68, 16, 8, 8, 5, 4, 2, 2
#6	7	15.57 ± 36.98	775, 135, 24, 9, 3, 2, 2
#7	6	13.97 ± 34.38	636, 143, 105, 57, 5, 4
#8	15	13.19 ± 30.04	662, 78, 59, 56, 23, 19, 18, 11, 9, 7, 4, 1, 1, 1, 1
#9	14	10.92 ± 14.45	294, 149, 132, 85, 81, 65, 53, 49, 14, 12, 9, 5, 1, 1
#10	10	12.18 ± 20.94	369, 226, 220, 68, 26, 25, 8, 6, 1, 1
#11	7	24.36 ± 43.06	591, 196, 112, 26, 20, 4, 1
#12	6	13.57 ± 24.68	506, 277, 77, 51, 38, 1
#13	15	7.98 ± 11.36	574, 147, 47, 39, 36, 34, 15, 15, 15, 12, 5, 5, 3, 2, 1
#14	11	12.18 ± 48.36	534, 140, 123, 53, 38, 36, 12, 7, 4, 2, 1
#15	16	10.78 ± 19.03	346, 296, 147, 65, 42, 11, 11, 5, 5, 5, 4, 3, 3, 3, 2, 2
#16	10	13.19 ± 25.68	839, 29, 23, 21, 16, 9, 8, 3, 1, 1
#17	5	45.24 ± 101.86	851, 74, 15, 9, 1
#18	14	10.88 ± 12.97	476, 116, 108, 96, 52, 34, 26, 11, 7, 6, 5, 5, 4, 4
#19	12	11.88 ± 22.20	659, 169, 25, 22, 15, 13, 13, 12, 8, 7, 6, 1
#20	9	27.14 ± 70.18	813, 84, 20, 17, 7, 4, 2, 2, 1
#21	10	25.00 ± 44.62	582, 326, 13, 11, 6, 4, 3, 2, 2, 1
Summary (mean ± standard deviation)	10.52 ± 4.05	13.47 ± 35.95	90.27 ± 177.96

**TABLE 2:** Summary of number of members and total occurrence times for third level clusters.

**Clusters**	**Number of members (dominant second level clustering patterns)**	**Total occurrence times (TRs)**
#1	3	789
#2	3	899
#3	2	910
#4	31	11,850
#5	4	796
#6	2	783
#7	2	721
Sum	47	16,748

Table 1 also conveys information about the number of second level clusters for each subject and the time spent before state (pattern) transition. Normally, the larger the number of clusters, the smaller the mean time (and its standard deviation) spent before a state transition. Their relationship is illustrated in Figure 5 using linear regression. The regression of mean time spent before state transition with respect to the number of clusters is shown in Figure 5A, and corresponding results for standard deviation of time spent before state transition are shown in Figure 5B.

**FIGURE 7:**
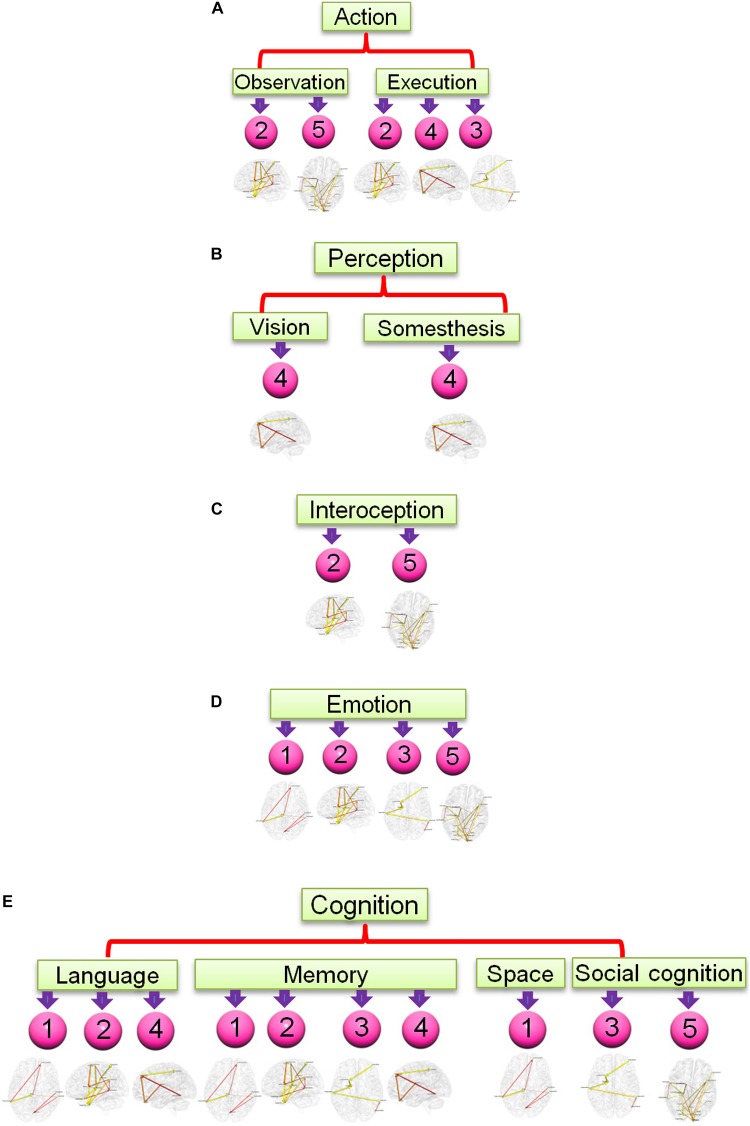
Functional relevance of the five networks obtained from the most consistent third level clustering centroid. The digits in pink balls mark the corresponding directional networks in Figure 6. The head nodes of networks indicate the functionality that co-activates the regions of corresponding networks as ascertained through activation likelihood estimation (ALE)-based meta-analyses using the BrainMap database. Flowchart **(A)** is for functionality of action, **(B)** is for perception, **(C)** is for interoception, **(D)** is for emotion, and **(E)** is for cognition.

The result for third level clustering is illustrated in Figure 6 and Table 2, and potential neural correlates of those connectivity patterns to real world functionalities are depicted in Figure 7. At the third level, seven clusters were found from all dominating second level clusters obtained from all subjects, but only the 4th cluster was dominant and consistent across all subjects (Table 2). Figure 6 visualizes the five first level clusters which make up the centroid of the 4th third level cluster. It is noted that at the first level, the number of clusters was set to 6, and one of those clusters (networks) was trivial because it included all other brain regions which were not present in the five other clusters. This happens because clustering partitions the input space, and if there are five definitive clusters, the 6th cluster will include everything that was excluded in the five clusters. In Figure 6, green dots represent the centers of the functionally homogeneous CC200 regions under consideration, and arrowed paths represent directional connectivities between regions with thickness and color indicating the absolute connectivity value. Network #1 in Figure 6A illustrates directional causal influences among right mid temporal area, left calcarine, left postcentral, and left inferior temporal area, as well as pons and left superior orbital frontal area. According to activation likelihood estimation (ALE)-based meta-analyses using the BrainMap database these regions are co-activated by emotional stimuli (Shapira et al., 2003), language processing (Fu et al., 2002), working memory (Sailer et al., 2007), and spatial information processing (Ricciardi et al., 2006). Network #2 in Figure 6B involves supplementary motor area (SMA), postcentral area, supramarginal area, mid temporal area, and thalamus in the right hemisphere, and insula, mid, and inferior temporal area, and precentral area in the left hemisphere. These regions are mostly distributed in parietal lobe, temporal lobe, as well as limbic regions. According to ALE-based meta-analyses using BrainMap, the co-activation of most of these regions is due to interoception (Karnath et al., 2005; Hu et al., 2008), working memory (Medaglia et al., 2012), language (Bookheimer et al., 2000), observation (Hu et al., 2008), execution (Bookheimer et al., 2000), as well as emotion regulation (Garrett and Maddock, 2006; Bokde et al., 2009). Besides the functionalities for areas above mentioned in Network #1, the SMA and precentral area are involved in movement control and execution (Ellermann et al., 1998); insula involved in emotion, perception, motor control, self-awareness, and interoception; postcentral area is involved in tactile sense. Network #3 shown in Figure 6C involves middle frontal area, inferior and mid temporal areas in the right hemisphere, and mid temporal area, caudate, and inferior orbital frontal area in the left hemisphere. We can clearly see the temporal to caudate causal pathway and frontal to caudate causal pathway. Caudate nucleus has been demonstrated to be involved in learning and memory (Graybiel, 2005), particularly regarding feedback processing (reward and motivation) (Kinnison et al., 2012), as well as emotion (Aron et al., 2005; Ishizu and Zeki, 2011). Together with temporal area and frontal area, these two pass ways indicate several functionalities using Brainmap Sleuth: memory (Malhi et al., 2007), execution, emotion (Lee et al., 2006), and social cognition (Strathearn et al., 2008). Network #4 shown in Figure 6D includes inferior parietal area, caudate, rectus, and superior frontal area in the right hemisphere, as well as left inferior temporal area. Rectal gyrus has been linked to attention and memory processing (Morecraft et al., 1992). Inferior parietal lobule is involved in interpretation of sensory input and perception of emotions. By using Brainmap Sleuth, the co-activation of most these areas was found to involve the functionalities of execution (Lissek et al., 2007), memory (Achim and Lepage, 2005), visual and somesthesis perception (Bingel et al., 2006), and language processing (Fu et al., 2002; Liljeström et al., 2008). Lastly, Network #5 in Figure 6E involves left SMA, left postcentral area, left supramarginal area, bilateral lingual gyrus, bilateral cuneus, superior bilateral occipital area, right fusiform, right inferior orbital and opercular frontal area, left parahippocampus gyrus, part of vermis, and left thalamus. Using Brainmap Sleuth, most of these areas are co-activated by emotion and social cognition (Strathearn et al., 2008), interoception and observation (Hu et al., 2008). Fusiform, superior occipital area, cuneus relate to visual perception and processing, lingual gyrus participates in visual processing and visual memory encoding, supramaginal relates to language processing, and postcentral area and parahippocampal gyrus relate to memory encoding and retrieval.

### Experimental Data (Cohort-2)

**FIGURE 8:**
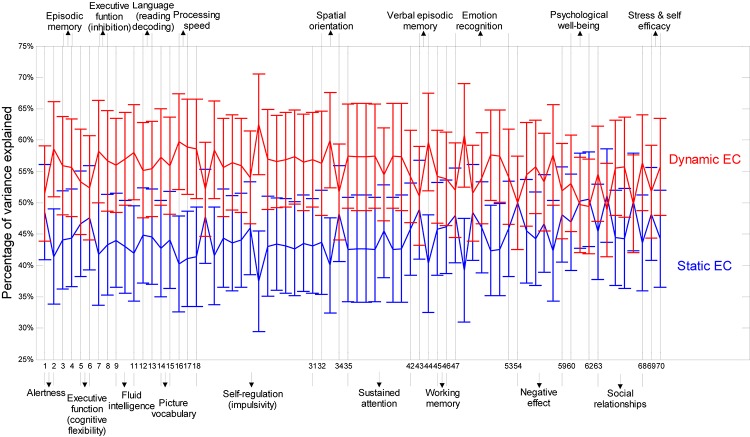
Percentage of variances in behavioral measures explained by dynamic and static EC metrics. Percentage of variances are shown as error bars with mean and standard deviation derived across all paths between the 190 regions. Along the horizontal axis are labels for 70 behavioral tests (refer to Table 3 for behavioral test details). The broad behavioral domains of groups of behavioral tests are indicated above and below the figure. It can be seen that the variances explained by dynamic EC metrics are distinctively higher than static FC for nearly each and every behavioral measure.

Figure 8 presents relative percentage of variance explained by static EC and dynamic EC for 70 behavioral scores such as alertness, cognition, emotion, and personality traits (refer to Table 3 for behavioral test details) obtained from Cohort-2. Noticeably, it is clear that the variances explained by dynamic EC metrics are distinctively higher than Static FC for nearly each and every behavioral measure. This implies that dynamic EC can be a better predictor of human behavior than conventional static EC. Scatter plots of behavioral measures against two metrics indicate that the correlations were not caused by outliers.

**TABLE 3:** Description of categorized behavioral measures employed in this work.

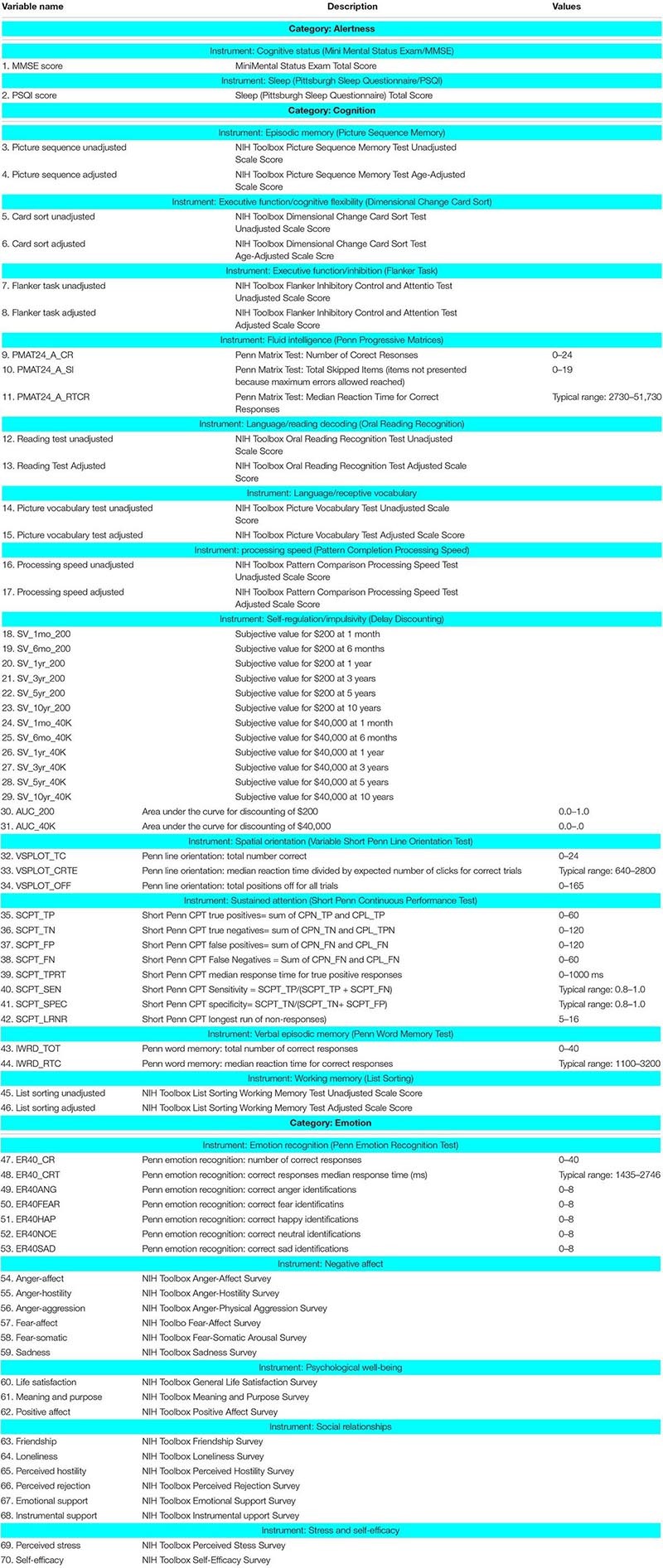

*For more details, refer to HCP Q3 release manual http://humanconnectome.org/documentation/Q3/Q3_Release_Reference_Manual.pdf.*

## Discussion

We propose a multi-level clustering algorithm for characterizing and understanding directional network patterns across spatial locations, time, and subjects. We validate the proposed method using simulations and demonstrate its utility using a dynamic EC measure. Further, we relate such clustering patterns to real-word functionalities using meta-analysis and demonstrate that dynamic EC explains more variance in behavioral measures across 70 different behavioral domains as compared conventional static EC. Below, we elaborate on major contributions of this work.

The alternating first level brain EC clusters or network patterns were subjected to second level clustering, and from this, we were able to identify one to three dominating patterns in individual subjects, with quasi-stable property. A dominating pattern will dominate for a period of time and during this period, no other dominating patterns exist. After this period, another dominating pattern takes over. During the period when one dominating pattern dominates, there may appear several sub-patterns, and like the dominating patterns, each sub-pattern will appear and alternate with the dominating one for only a section of time, and after this time, another sub-pattern takes over. Then, under the sub-patterns, there may appear even smaller sub-patterns which behave similarly to sub-patterns, but at a finer scale. Previous reports have found this fractal-like quasi-stable phenomenon using EEG/fMRI analysis (Britz et al., 2010; Musso et al., 2010; Van de Ville et al., 2010). Both functional connectivity networks and the topography of the spontaneous EEG show stable global brain states remaining quasi-stationary for a period of time, called microstates. Our results suggest that quasi-stable EC network configurations may support or be related to brain microstates. Further study is necessary to investigate this phenomenon and is one of our future research directions.

At the third level, we obtained seven clusters and among them, we found one dominating and consistent EC network pattern across all subjects. Detailed analysis of all networks in this pattern demonstrates that there are five EC networks in this pattern, and these networks contain several regions: mid and inferior temporal cortex, frontal cortex, SMA, pre and postcentral area, parietal cortex, and occipital cortex. These areas are frequently recruited when the brain is engaged in memory retrieval, observation, execution, and emotion regulation. These cognitive functions are most frequently encountered for resting-state human brain when lying in scanner. Other regions such as caudate, hippocampus, fusiform, and lingual cortex are also implicated in above functions (Bogousslavsky et al., 1987; Bliss and Collingridge, 1993; Grahn et al., 2008). The language function and execution function involve three networks which is less than memory, and emotion. This may be due to the reason that language and execution processes are not as frequently recruited as the other three at rest. Functionalities including observation, somesthesis, vision, interoception, spatial cognition, and social cognition involve even less networks maybe due to the reason these functions are less frequently employed when lying rest during fMRI scan.

It must be noted that three levels of clustering is required to find patterns consistent across three varying factors: time, spatial location, and subjects. However, it is not necessary in all cases. For example, if only patterns across time are of interest, a single level AEC across time will suffice. There are multiple studies adopting *K*-means or hierarchical clustering across spatial locations to find brain networks. Our point is that single level clustering has always existed and we are proposing multi-level clustering for estimating consistent patterns across different varying factors (such as time, spatial location, and subjects). The framework could also be extended to have more than three levels of clustering if someone wants to investigate consistent patterns across more than three varying factors.

Previously we investigated behavioral relevance of static and dynamic functional connectivity (DFC) (Jia et al., 2014). Since EC has been shown to be a complimentary mode of communication between brain regions in resting state, we used a similar framework for dynamic and static EC metrics here. Akin to our previous study, the results from the current study demonstrate that dynamic EC is able to explain more variance in behavioral performance tasks compared to SEC metric for a total of 70 behavioral tests included, which contains domains such as alertness, cognition, emotion, and personality. This supports our hypothesis that greater temporal variability increases the adaptability of brain networks, leading to better behavioral performance.

Admittedly, this work suffers from several drawbacks. First, the data did not cover cerebellum such that this brain region was not considered in the analysis. For future work, using resting-state data covering the whole brain for analysis of this kind is necessary. On the other hand, the switching pattern of brain network configurations in this work is similar to that of EEG microstates that have been shown to have fractal property (Van de Ville et al., 2010). In the future, a similar analysis using simultaneous EEG–fMRI data may allow for a comparison of quasi-stable network patterns obtained from EEG and fMRI.

## Data Availability Statement

The datasets generated for this study are available on request to the corresponding author.

## Ethics Statement

The studies involving human participants were reviewed and approved by the IRB at University of Minnesota (for HCP data) and the IRB at Auburn University (for in-house MRI data). The patients/participants provided their written informed consent to participate in this study.

## Author Contributions

GD conceived the idea in consultation with HJ. HJ implemented the idea under guidance from GD. HJ wrote the first draft of the manuscript and GD substantially added and edited it.

## Conflict of Interest

The authors declare that the research was conducted in the absence of any commercial or financial relationships that could be construed as a potential conflict of interest.

## Footnotes

1 https://www.humanconnectome.org/storage/app/media/documentation/q3/Q3_Release_Reference_Manual.pdf

2 www.nihtoolbox.org
